# An Annotation-Free Pipeline for 3D Auricular Bowl Atlas Construction and Statistical Shape Modelling from Surface Scans

**DOI:** 10.3390/s26113493

**Published:** 2026-06-01

**Authors:** Tongxu Zhang, Tony Kwok Wing Lee, Jiebin Huang, Kam Lun Leung, Siu Ngor Fu

**Affiliations:** Department of Rehabilitation Sciences, The Hong Kong Polytechnic University, Kowloon, Hong Kong, China; jukie.zhang@connect.polyu.hk (T.Z.); kwok-wing-tony.lee@connect.polyu.hk (T.K.W.L.); jiebin.huang@connect.polyu.hk (J.H.); amy.fu@polyu.edu.hk (S.N.F.)

**Keywords:** 3D scanning, 3D ear morphology, statistical shape model, surface registration, thin-plate spline, point cloud

## Abstract

Three-dimensional (3D) ear morphology is critical for the design of in-the-ear hearing aids, earphones, transcutaneous auricular vagus nerve stimulation (taVNS) electrodes, and auricular reconstruction, yet most existing ear shape models still rely on manually placed landmarks. Here, a fully annotation-free pipeline is presented for constructing a 3D ear atlas and statistical shape model (SSM) of the auricular bowl from 50 surface meshes. Individual ears are iteratively registered to a current atlas using rigid the iterative closest point (ICP) algorithm followed by a bidirectional thin-plate spline (BiTPS) deformation, and dense surface correspondences are established by nearest-neighbour mapping. Registration quality is quantified using mean and maximum nearest-neighbour distance, symmetric Chamfer-$L_{2}$ distance and coverage. Furthermore, SSM-derived bowl height and width are validated against manual 3D mesh measurements in Geomagic Design X. Across five atlas iterations, the BiTPS pipeline substantially reduces registration errors and increases coverage, and principal component analysis (PCA) derived dimensions show excellent agreement with manual measurements (Pearson r≥0.98, ICC ≥0.98). The proposed framework yields a stable, anatomically plausible ear atlas and an interpretable low-dimensional SSM without manual landmarks, providing a computational basis for the geometric optimization of ear-related medical and wearable devices.

## 1. Introduction

A growing number of in-ear and periauricular medical devices—including hearing aids [1], auricular acupressure [2] and transcutaneous auricular vagus nerve stimulation (taVNS) electrodes [3,4]—rely on intimate contact with the auricular bowl region to achieve stable coupling, effective stimulation, and long-term comfort. In parallel, surgical auricular reconstruction, orthotic ear moulding in infancy, and prosthetic auricular rehabilitation depend on a precise understanding of the three-dimensional (3D) morphology of the external ear to restore a natural appearance [5,6,7], while providing adequate good contact and support areas [8,9]. Poor geometric compatibility in this region can be associated with pressure points, pain, loss of acoustic sealing, or unstable electrical contact [10].

Traditional auricular acupressure—utilizing Vaccaria seeds or magnetic pellets—relies on adhesive tape, which is prone to causing dermatological irritation and requires frequent, lobar-intensive replacement. While traditional earbuds rely on the ear canal for retention and taVNS electrodes are typically positioned within the cavum and cymba conchae [11,12,13,14], this study aims to develop a basic structure for the design of an auricular acupressure splint that incorporates acupoints on the conchae, triangular fossa, and antihelix. This design applies targeted mechanical pressure to modulate physiological functions [15,16,17]. In addition to housing the acupoints, the triangular fossa and antihelix serve as the primary anatomical anchors for the splint.

Two-dimensional (2D) auricular acupoint maps are traditionally used to locate acupoints for stimulation [18]. Historically, the quantification of auricle morphology in clinical and industrial design has been limited to a few linear measurements or has relied on experienced practitioners. Furthermore, recent advances in 3D scanning and statistical shape analysis [19,20,21,22] enable a more detailed characterisation of ear morphology for ergonomic design, perceived fit, and comfort [10,20,21]. Additionally, deep learning-based point cloud reconstruction and upsampling methods have been proposed to generate high-fidelity 3D medical shapes from sparse or noisy inputs [23,24].

Most existing ear shape modelling approaches rely on manually labelled landmarks (dense or sparse), with correspondences subsequently propagated through non-rigid registration [21,25]. However, given the complex anatomical structures and indistinct boundaries of the auricle, reliable landmark placement is time-consuming and may suffer from limited inter-operator reliability.

To avoid reliance on manual landmarks, an annotation-free 3D ear statistical modelling method was proposed. A population-average ear, or “atlas”, was iteratively constructed, and individual auricles were registered to the current atlas using rigid-body iterative closest point (ICP) [26,27] and non-rigid bidirectional thin-plate spline (BiTPS) deformations [28,29]. Dense surface correspondence was established through nearest-neighbour search. Following the convergence of the atlas, principal component analysis (PCA) [30] was performed on point clouds with one-to-one correspondence to obtain a population-wide ear statistical shape model (SSM) [22]. This model could be interpreted within the commonly used framework of ear height, width, depth, and conchal morphology. Specifically, “annotation-free” denotes that no manually annotated anatomical landmarks were required; instead, the BiTPS control points were automatically selected by farthest point sampling (FPS) on the atlas.

Our methodological novelty lies not in the introduction of a new deformation model, but rather in providing an engineering-ready, annotation-free end-to-end atlas/SSM pipeline with transparent evaluation and reproducible correspondence construction for the auricular bowl.

This research contributes a validated, region of interest (ROI)-specific atlas designed for the geometric optimization of auricular acupressure splints and ear-wearable devices. By establishing the “auricular bowl” as a standardized design space, we provide a foundation for downstream clinical and industrial applications, such as automated acupoint localization, which were previously limited by the absence of a unified statistical model.

The primary technical contributions of this work are as follows:

1. An automated, annotation-free SSM pipeline: We present an end-to-end framework for atlas and statistical shape model (SSM) construction specifically for the auricular bowl. By leveraging automatically selected pseudo-landmarks via farthest point sampling (FPS) for deformation parametrisation, the approach eliminates the need for labour-intensive manual anatomical annotation.
2. Systematic evaluation of registration evolution: We provide a rigorous assessment of registration quality and atlas convergence across iterative stages. By comparing a rigid-only baseline against non-rigid BiTPS refinements, we demonstrate the specific alignment improvements and convergence behaviour of the model.
3. Metrological validation for practical application: Beyond qualitative shape analysis, we establish the model’s reliability through a comparative study of model-derived versus manual 3D mesh measurements. Using Bland–Altman analysis and the Intraclass Correlation Coefficient (ICC), we demonstrate the high metrological agreement necessary for practical sizing and fit optimization in wearable design.

## 2. Methods

**Figure 1:**
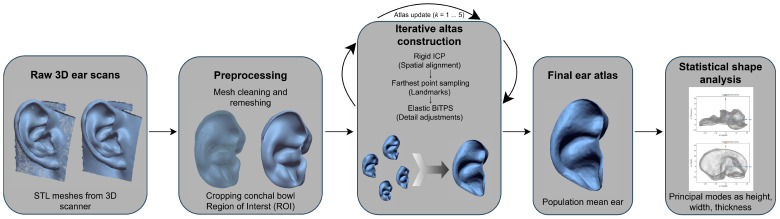
Overview of the proposed annotation-free 3D ear atlas and statistical shape model (SSM) pipeline. Raw 3D ear scans were first cleaned, cropped to the auricular bowl region, and remeshed (preprocessing). Individual ears were then iteratively registered to a current atlas using rigid ICP and non-rigid BiTPS, yielding a refined population mean ear (final ear atlas). Finally, a statistical shape model was constructed via PCA on the aligned meshes, and the principal modes of variation were interpreted in terms of ear height, width, and bowl morphology.

An overview of the proposed annotation-free 3D ear atlas and statistical shape model (SSM) pipeline is shown in Figure 1. Following the acquisition of raw 3D ear meshes, mesh cleaning and cropping were performed. Subsequently, iterative atlas construction was carried out using rigid ICP and non-rigid BiTPS, followed by PCA-based statistical shape analysis on the aligned meshes. The pseudocode for the complete process is provided in Algorithm 1.
**Algorithm 1:** Annotation-free atlas construction and SSM for the auricular bowl
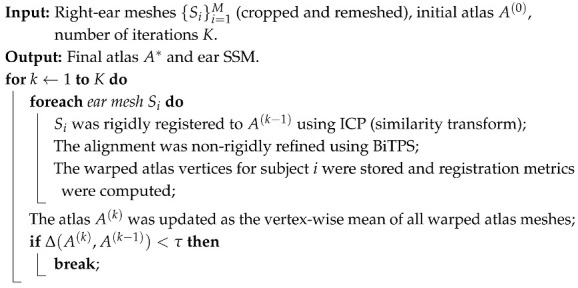


### 2.1. Data Acquisition and Preprocessing

#### 2.1.1. Participants and Ethics

A total of 50 right ears from 50 participants were included in this study (comprising 25 males and 25 females; age range 13–45 years). Written informed consent was obtained from all participants. The study protocol was approved by the institutional ethics committee of The Hong Kong Polytechnic University (approval ID: HSEARS20230705002). All procedures were conducted in accordance with the Declaration of Helsinki.

#### 2.1.2. 3D Scanning

All ears were captured using an EinScan Pro HD structured-light scanner (Shining 3D), with an effective spatial resolution of approximately 0.2 mm. For each subject, the right auricle region was scanned with the head in a neutral position. Raw meshes were exported in STL format for subsequent processing (Figure 1).

#### 2.1.3. Mesh Cleaning and Cropping

The raw meshes were denoised and cleaned using *Geomagic Design X* (version 2024.3, Oqton Inc., San Francisco, CA, USA), which included the removal of isolated components, spike filtering, and hole filling where appropriate. To obtain a consistent surface representation, each ear mesh was isotropically remeshed to approximately $4\times 10^{4}$ to $6\times 10^{4}$ triangles using the Decimate function in *Blender* (version 4.0, Blender Foundation, Amsterdam, The Netherlands).

Among them, to minimize occlusion in the concave regions of the conchae, each subject was scanned from multiple perspectives. Minor surface voids were processed using a curvature-based hole-filling algorithm (Shining 3D), and the resulting meshes were manually inspected to ensure no structural distortion occurred within the core ROI.

### 2.2. Geometry and Surface Sampling

#### Triangle-Area-Weighted Sampling

To obtain a uniform point cloud representation, each ear surface was sampled proportionally to the triangle area. For a triangular face with the vertices $v_{0},\;v_{1},$ and $v_{2}$, the area was calculated as follows:

(1) $$A_{i}=\frac{1}{2}∥\left(v_{1}-v_{0}\right)\times \left(v_{2}-v_{0}\right)∥,$$

where $A_{i}$ denotes the area of the *i*-th triangle.

The areas were normalized to obtain sampling probabilities:

(2) $$p_{i}=\frac{A_{i}}{\sum_{j}A_{j}},$$

where $p_{i}$ denotes the probability of sampling from the *i*-th triangle.

Barycentric coordinates were randomly generated inside each triangle. Specifically, (u, v) were drawn from a uniform distribution on ${\left[0,\;1\right]}^{2}$ and mapped as follows:

(3) $$\left(u^{\prime },\;v^{\prime },\;w^{\prime }\right)=\left\{\begin{array}{cc} (u,\;v,\;1-u-v),\; & u+v\leq 1,\\ (1-u,\;1-v,\;u+v-1), & u+v>1, \end{array}\right.$$

where $\left(u^{\prime },\;v^{\prime },\;w^{\prime }\right)$ are the barycentric coordinates used for sampling points within the triangle.

The sampled point was then computed as

(4) $$x=u^{\prime }v_{0}+v^{\prime }v_{1}+w^{\prime }v_{2}.$$

This approach yields points that were approximately uniformly distributed with respect to surface area. For all subsequent registration and SSM stages, each mesh was represented by a fixed-size, uniformly sampled point set. In this study, a set of 20,000 points was used for each subject, thereby eliminating the requirement for identical vertex counts across the original raw meshes.

### 2.3. Iterative Atlas Construction

Let ${\left\{S_{i}\right\}}_{i=1}^{M}$ denote the set of *M* training ear surfaces after sampling. A population atlas was constructed by iteratively registering all ear surfaces to an evolving template and averaging the aligned shapes.

#### 2.3.1. Initial Atlas

The atlas $A^{\left(0\right)}$ was initialized as a single ear surface from the training set that was representative in terms of overall size and appearance. In practice, the final atlas was found to be insensitive to the exact choice of the initial subject.

#### 2.3.2. Outer Iterations

For iteration k=1, …, K (here K=5), the following steps are performed:

1. *Rigid alignment.* Each ear $S_{i}$ was first pose-normalized by centring and enforcing a consistent global orientation, and it is then rigidly aligned to the current atlas $A^{\left(k-1\right)}$ using ICP with Umeyama’s closed-form transform. Depending on the scale setting, the Umeyama step estimates either rotation or translation only, so it will keep the scale factor, resulting in a rigidly aligned surface ${\tilde{S}}_{i}^{\left(k\right)}$.
2. *Optional non-rigid refinement.* In the BiTPS setting, a thin-plate spline (TPS) with bidirectional deformation was fitted between the atlas and ${\tilde{S}}_{i}^{\left(k\right)}$, yielding a non-rigidly aligned surface ${\hat{S}}_{i}^{\left(k\right)}$. In the pure ICP setting, ${\hat{S}}_{i}^{\left(k\right)}$ is set to ${\tilde{S}}_{i}^{\left(k\right)}$.
3. *Correspondence update.* Using nearest-neighbour mapping from the atlas to each ${\hat{S}}_{i}^{\left(k\right)}$, a corresponding point on each subject was obtained for every atlas point.
4. *Atlas update.* A new atlas $A^{\left(k\right)}$ was computed by averaging the corresponding points over all subjects:
  (5) $$A^{\left(k\right)}\left(p_{j}\right)=\frac{1}{M}\sum_{i=1}^{M}{\hat{S}}_{i}^{\left(k\right)}\left(p_{j}\right),$$
  where $p_{j}$ indexes atlas vertices.

This iterative process was executed in two separate configurations: first using rigid ICP only, and subsequently using ICP followed by non-rigid BiTPS registration. Registration metrics were recorded at each iteration to facilitate convergence analysis. Across iterations, a mild decrease in the atlas scale was observed—a phenomenon known as scale drift—during the template update phase under nearest-neighbour correspondence. When the rigid step can be configured to estimate a global scale factor, the observed drift during atlas updates was not solely determined by this setting. To correct for this and ensure consistent reporting of absolute measurements, the final atlas was rescaled after convergence to match the mean ear size of the training set. Registration metrics were recorded at each iteration to enable convergence analysis. Across iterations, we observed a mild decrease in the atlas scale due to scale drift bias, during mean-template updating under the nearest-neighbour correspondence. When the scaling-enabled setting is used, the rigid step can additionally estimate a global scale factor; however, the observed drift during atlas updates is not solely determined by this option. After convergence, the final atlas was re-scaled to match the mean ear size of the training set to facilitate consistent reporting of absolute measurements.

### 2.4. Rigid ICP with Umeyama Scale Estimation

#### 2.4.1. Closed-Form Umeyama Registration

Given two corresponding point sets $X={\left\{x_{i}\right\}}_{i=1}^{n}$ and $Y={\left\{y_{i}\right\}}_{i=1}^{n}$, a similarity transform (s, R, t) is sought that minimizes

(6) $$\sum_{i=1}^{n}∥y_{i}-\left(sRx_{i}+t\right){∥}^{2},$$

where R∈SO(3) is a rotation, s>0 is a global scale, and $t\in R^{3}$ is a translation. Let $\mu_{x}$ and $\mu_{y}$ denote the centroids of *X* and *Y*, and let centred coordinates be defined as $\bar{X}=X-\mu_{x}$ and $\bar{Y}=Y-\mu_{y}$. The covariance was given by

(7) $$C=\frac{1}{n}{\bar{X}}^{⊤}\bar{Y}.$$

An SVD $C={USV}^{⊤}$ was performed and *R* was set to ${VU}^{⊤}$; if det(R)<0, the sign of the last column of *V* was flipped before forming *R*. Scale was included so that the optimal scale and translation were given by

(8) $$s=\frac{\mathrm{tr}(S)}{\mathrm{var}(X)},\;t=\mu_{y}-sR\mu_{x},$$

where $\mathrm{var}\left(X\right)=\frac{1}{n}\sum_{i=1}^{n}{∥x_{i}-\mu_{x}∥}^{2}$ and $\mu_{x}$ and $\mu_{y}$ denote the means of the point sets *X* and *Y*, respectively.

#### 2.4.2. ICP Iteration

Given a source point cloud *X* and a target point cloud *Y*, the ICP algorithm iteratively alternated between among three steps:

1. *Correspondence.* Nearest-neighbours $Y^{\prime }$ are identified for the current transformed source $X^{\prime }$.
2. *Alignment.* The similarity transform parameters $\left(s_{i},\;R_{i},\;t_{i}\right)$ are estimated between $X^{\prime }$ and $Y^{\prime }$ using Umeyama’s method.
3. *Update.* The transformed source is updated as $X^{\prime }\leftarrow s_{i}R_{i}X^{\prime }+t_{i}$, and the accumulated transforms are updated according to
  (9) $$R\leftarrow R_{i}R,\;t\leftarrow s_{i}R_{i}t+t_{i},\;s\leftarrow s\;\cdot \;s_{i}.$$

The iteration was terminated when the decrease in the mean squared nearest-neighbour distance fell below a predefined tolerance. The resulting similarity transform was then applied to the original vertices. In this configuration used here, the scale factor was fixed at s=1 to preserve the absolute dimensions of the subjects.

### 2.5. Template Landmarks and Sample Projection

#### 2.5.1. Template Landmarks via FPS

To drive the non-rigid registration, *m* uniformly distributed template landmarks *Q* were selected from the atlas using farthest point sampling (FPS). In this procedure, the vertex farthest from the existing set of the selected set is iteratively added to ensure optimal surface coverage.

#### 2.5.2. Sample Correspondence

To establish correspondences, for each rigidly aligned sample surface ${\tilde{S}}_{i}$, the surface was represented by a dense point cloud of 20,000 points. For each landmark q∈Q, its nearest-neighbour *p* on the sample is found. The resulting point pairs (q, p) were then utilized as control points for non-rigid BiTPS registration.

### 2.6. Bidirectional Thin-Plate Spline (BiTPS) Registration

#### 2.6.1. Kernel and Linear System

A 3D thin-plate spline (TPS) model is used with kernel $U\left(r\right)=r^{2}\log r$, with U(0)=0. Given control points $X\in R^{n\times 3}$ and target points $Y\in R^{n\times 3}$, pairwise distances are defined as

(10) $$r_{ij}=∥x_{i}-x_{j}∥,$$

and the kernel matrix is constructed as

(11) $$K_{ij}=r_{ij}^{2}\log\left(r_{ij}+\varepsilon \right),$$

where ε is a small constant to avoid numerical issues. Let P=[1, X] denote the affine design matrix. The TPS parameters are obtained by solving

(12) $$\left[\begin{array}{cc} K+\lambda I & P\\ P^{⊤} & 0 \end{array}\right]\left[\begin{array}{c} W\\ A \end{array}\right]=\left[\begin{array}{c} Y\\ 0 \end{array}\right],$$

where λ (smooth) is a Tikhonov regularisation parameter. For query points *Q*, the deformation is given by

(13) $$f\left(Q\right)=K\left(Q,\;X\right)W+P_{Q}A.$$

#### 2.6.2. Bidirectional Mapping

Both the directions Q→P (template → sample) and P→Q (sample → template) were fitted. The former was used to warp the atlas vertices and to obtain non-rigid correspondences $V_{\mathrm{corr}}$, while the latter was used for evaluation of surface-based metrics such as coverage and Chamfer distance. This bidirectional TPS registration scheme was referred to as BiTPS.

## 3. Evaluation

### 3.1. Matching Quality and Coverage Metrics

To quantify registration quality between an aligned ear surface $S_{i}$ and the atlas *A*, the following metrics were used:

- **Chamfer-L_2_ [31,32].** The symmetric squared Chamfer distance between two point sets *A* and *B* is defined as
  (14) $$\mathrm{Chamfer}\left(A,\;B\right)=E_{a\in A}∥a-{\mathrm{NN}}_{B}\left(a\right){∥}^{2}+E_{b\in B}∥b-{\mathrm{NN}}_{A}\left(b\right){∥}^{2},$$
  where ${\mathrm{NN}}_{B}\left(a\right)$ denotes the nearest-neighbour of *a* in *B*. In practice, this is implemented using KD-tree nearest-neighbour search.
- **Coverage_*τ*_ [33,34].** Coverage is defined as the fraction of template points whose nearest-neighbour distance to the warped sample is below a threshold τ:
  (15) $${\mathrm{Coverage}}_{\tau }\left(A,\;B\right)=\frac{1}{\left|A\right|}|\{a\in A:∥a-{\mathrm{NN}}_{B}\left(a\right)∥\leq \tau \}|.$$
- **Mean and maximum nearest-neighbour distances [35,36].** For more detailed assessment, the mean nearest-neighbour distance was reported as
  (16) $$\mathrm{mean\_nn}\left(A,\;B\right)=E_{a\in A}∥a-{\mathrm{NN}}_{B}\left(a\right)∥,$$
  and the corresponding maximum distance was given by
  (17) $$\mathrm{max\_nn}\left(A,\;B\right)=\max_{a\in A}∥a-{\mathrm{NN}}_{B}\left(a\right)∥.$$

These metrics were recorded for each subject, for both ICP and BiTPS pipelines, and at each atlas iteration.

### 3.2. Statistical Shape Model (PCA)

After convergence of the atlas, all aligned meshes were brought into dense point-to-point correspondence. Each mesh $V_{m}\in R^{N\times 3}$ was reshaped into a vector of length 3N, and a data matrix $X\in R^{M\times 3N}$ was formed over *M* subjects. After subtraction of the mean μ, singular value decomposition (SVD) was performed as

(18) $$\frac{1}{\sqrt{M-1}}X_{c}={USV}^{⊤},$$

where $X_{c}$ denotes the centred data matrix. The variances along the principal components were given by $\sigma_{i}^{2}=S_{i}^{2}$. The top *K* modes that explained 99% of the total variance were retained (i.e., *K* is determined automatically by the 99% cumulative-variance criterion). The mean shape was reshaped back to (N, 3), and the top *K* principal components were reshaped to (K, N, 3) for visualisation. To illustrate variability, shapes corresponding to $\mu \pm 2\sqrt{\sigma_{i}^{2}}$ along each mode were rendered.

To evaluate reconstruction performance, each shape was projected onto the retained subspace and reconstructed using the mean plus the first *k* modes, where *k* was swept from 0 to *K* to generate reconstruction error curves as a function of the included components.

### 3.3. Manual Measurements and Statistical Analysis

#### 3.3.1. Manual Measurements with Standardized Extremal Landmarks

Manual reference measurements were obtained on the cleaned and cropped 3D auricle meshes using in *Geomagic Design X* (version 2024.3, Oqton Inc.). To ensure consistency and to eliminate dependence on arbitrary viewing angles, each mesh was first transformed into the standardized atlas coordinate system. This step utilized the same rigid alignment parameters established during the atlas construction, such that the superior–inferior (SI) and anterior–posterior (AP) axes were consistently oriented across all subjects.

**Figure 2:**
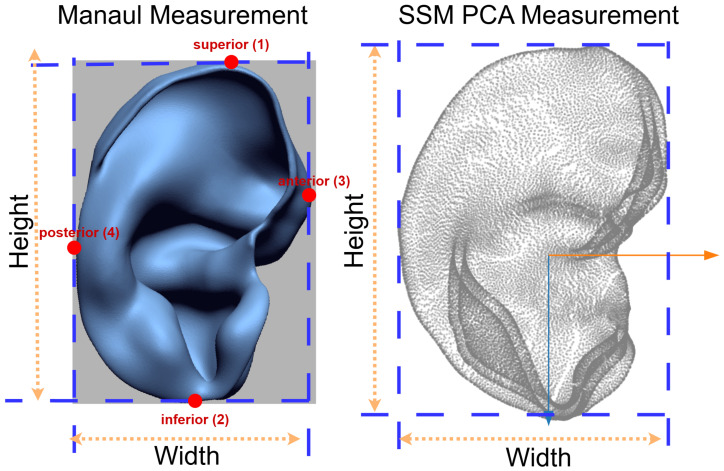
Manual and SSM/PCA-based definitions of ear height and width on the lobule-excluded auricular bowl region. After alignment to the atlas coordinate system, height was defined as the superior–inferior extent of the cropped auricle and width was defined as the anterior–posterior extent. The red markers indicate the superior (1), inferior (2), anterior (3), and posterior (4) extremal points within the cropped region. The same coordinate axes and geometric definitions were applied to the sampled atlas point cloud and SSM/PCA reconstructions to ensure consistency with the manual measurements in *Geomagic Design X*.

In accordance with standard anthropometric definitions [37,38] of *ear height (length)* and *ear width (breadth)*, the required landmarks were operationalized as *xtremal points* within the ROI. As illustrated in Figure 2, this ROI corresponds to the auricular bowl, with the lobule explicitly excluded.

Specifically, four extremal landmarks were defined on the aligned ROI:

- **SEP (superior extremal point)**: the most superior point of the ROI, defined by the maximum coordinate along the superior–inferior axis.
- **IEP (inferior extremal point)**: the most inferior point of the *lobule-excluded* ROI boundary in minimum SI coordinate, defined by the minimum coordinate along the superior–inferior axis.
- **PEP (posterior extremal point)**: the most posterior point of the ROI (minimum anterior–posterior coordinate), which serves as the functional equivalent to the *posterior auricle* landmark used in full-pinna anthropometry.
- **AEP (anterior extremal point)**: the most anterior point of the ROI (maximum anterior–posterior coordinate). This point serves as a functional surrogate of the anterior outer-rim landmark used in ear-breadth definitions, which is typically located near the anterior helix or root region in full-pinna studies.

Based on these standardized extremal landmarks, *ear height (length)* and *ear width (breadth)* were computed as axis-aligned distances:

(19) H=y(SEP)−y(IEP),W=x(AEP)−x(PEP),

where y(·) and x(·) denote the coordinates along the superior–inferior and anterior–posterior axes within the standardized coordinate frame. Although the ROI excludes the lobule, the IEP represents the inferior extremal point of the cropped auricular bowl rather than the anatomical lobule tip. Nevertheless, the same cropping protocol was applied consistently across subjects, ensuring a reproducible and standardized measurement. To minimize inter-observer variability, all measurements were performed by a trained practitioner using an identical protocol across subjects.

#### 3.3.2. Model-Derived Measurements

The same morphometric dimensions were computed for the atlas and the SSM-reconstructed ears. For each subject, the original mesh was reconstructed from the SSM using all *K* retained principal components. Ear height, ear width, and concha height were then extracted from reconstructed meshes along the predefined anatomical axes, utilizing the same geometric definitions applied during the manual measurement process in Geomagic. This protocol yielded paired measurements (manual vs. SSM-derived) for each dimension across all subjects, enabling a direct evaluation of the model’s geometric fidelity.

#### 3.3.3. Agreement Between Manual and Model Measurements

For each dimension (ear height, ear width, and concha height), association between manual ground-truth and SSM-derived measurements was assessed using Pearson’s correlation coefficient (*r*) for linear relationships and Spearman’s rank correlation coefficient (ρ). To assess systematic bias and quantity measurement error, the differences ($d_{i}$) were computed as

(20) $$d_{i}={\mathrm{SSM}}_{i}-{\mathrm{manual}}_{i},$$

and paired *t*-tests (or Wilcoxon signed-rank tests where the assumption of normality was violated) were applied to assess the significance of the differences. The mean difference ($\bar{d}$), standard deviation (SD), and 95% confidence intervals (CI) were reported. Bland–Altman plots were constructed to visualise the mean difference (systematic bias) and the 95% limits of agreement (LoA) (mean ±1.96 SD). Overall agreement was further quantified using the ICC based on a two-way mixed-effects model, absolute agreement, and a single-rater measurement (ICC(A,1)), as is standard for method-comparison studies. All statistical analyses were performed using Python (version 3.10).

#### 3.3.4. Analysis of Iterative Registration Metrics

To evaluate the influence of the outer atlas iterations, registration metrics including nearest-neighbour distance (mean_nn), maximum nearest-neighbour distance (max_nn), Chamfer-L_2_ distance, and coverage (${\mathrm{Coverage}}_{\tau }$) were analysed across all iterations for both ICP and BiTPS pipelines. For each metric, a repeated-measures design was employed, comprising one observation per subject across each of the K=5 outer atlas iterations.

#### 3.3.5. Analysis of SSM Compactness, Variance and Reconstruction Accuracy

Following established protocols in statistical shape modelling [39], the resulting SSM was evaluated based on its compactness, generalization, and reconstruction accuracy.

Compactness assesses the efficiency with which the model represents population variability. This was quantified by calculating the variance of the principal component (PC) scores across all subjects, expressed as both the individual and cumulative fractions of total variance explained by the leading modes. The resulting variance curves indicate the amount of shape information captured as the number of retained PCs increases.

To further characterise inter-individual variability within the learned shape space, we examined the cumulative fraction of total variance explained by the leading principal components (PCs). We then visualised the organization of subject-level variation using scatter plots of scores along the first two PCs (PC1 and PC2).

To assess reconstruction accuracy as a function of SSM dimensionality, each aligned mesh was projected onto the principal subspace and reconstructed using the mean shape plus the first *k* principal components, with *k* ranging from 0 (mean shape only) to the full number of retained modes. For each subject and each *k*, the vertex-wise root-mean-square error (RMSE) and the symmetric Chamfer-L_2_ distance between the original and reconstructed meshes were computed. The mean errors across all 50 ears were then summarised as functions of *k* to characterise the trade-off between model compactness and geometric fidelity.

## 4. Results

### 4.1. Registration Accuracy and Atlas Evolution Across Iterations

**Figure 3:**
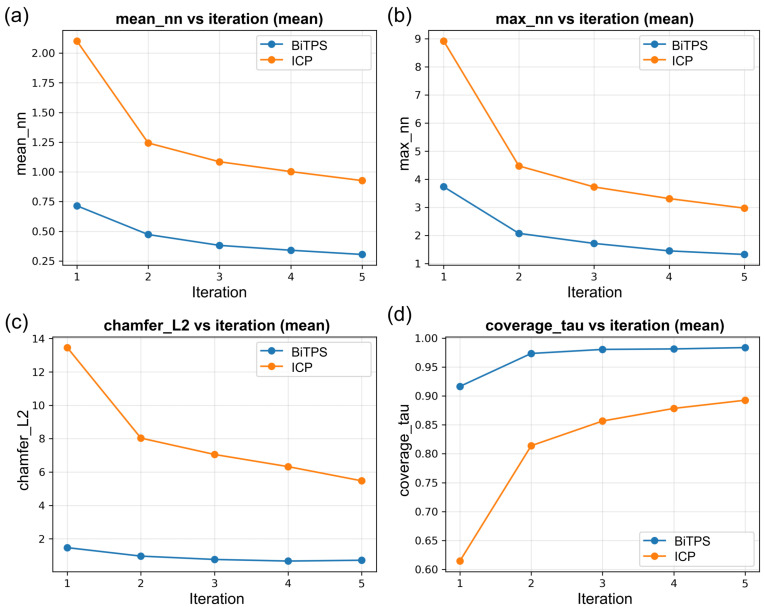
Registration metrics as a function of atlas iteration for rigid ICP and non-rigid BiTPS (mean across 50 ears). (**a**) Mean nearest-neighbour distance mean_nn; (**b**) maximum nearest-neighbour distance max_nn; (**c**) Chamfer-L_2_ distance; (**d**) ${\mathrm{Coverage}}_{\tau }$.

The progression of registration quality over the K=5 outer atlas iterations was first analysed for both the rigid ICP and non-rigid BiTPS pipelines. Figure 3 summarises the mean registration metrics across all 50 ears as a function of iteration, with key values at iterations 1 and 5 provided in Table 1. For the rigid ICP pipeline, the mean nearest-neighbour distance mean_nn decreased from 2.10 at iteration 1 to 0.93 at iteration 5, while the maximum nearest-neighbour distance max_nn was reduced from 8.92 to 2.97. The mean Chamfer-L_2_ distance showed a similar trend, decreasing from 13.46 to 5.47. Concurrently, the coverage metric ${\mathrm{Coverage}}_{\tau }$ increased from 0.61 to 0.89, indicating that an increasing fraction of atlas points achieved valid correspondences with the target auricular surfaces as the refinement progressed.

A comparable pattern of improvement was observed for the BiTPS pipeline, although substantially lower error magnitudes were achieved at all iterations. The mean mean_nn was reduced from 0.71 to 0.30, while max_nn decreased from 3.73 to 1.32 between iterations 1 and 5. Similarly, the mean Chamfer-L_2_ distance was reduced from 1.47 to 0.71, while ${\mathrm{Coverage}}_{\tau }$ increased from 0.92 to 0.98, nearly reaching complete coverage of the atlas surface. For both ICP and BiTPS pipelines, significant iteration effects were observed across all metrics (Friedman tests, $\chi_{\left(4\right)}^{2}\geq 152.8$, $p<10^{-31}$), confirming that registration accuracy improved systematically over the five atlas updates.

**Figure 4:**
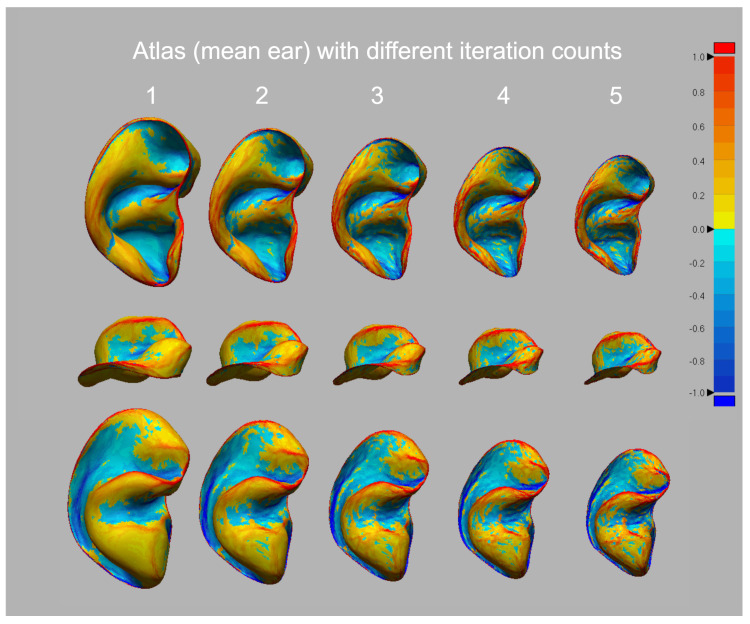
Evolution of the BiTPS atlas (mean ear) over outer iterations, visualised with surface curvature. Columns 1–5 correspond to atlas iterations k=1, …, 5. Across iterations, local curvature patterns remain stable while the global scale of the atlas gradually decreases, reflecting improved anatomical alignment without distortion of fine folds. The final atlas is rescaled to match the mean ear dimensions of the training set for subsequent statistical analysis.

The corresponding evolution of the atlas geometry and curvature is visualised in Figure 4. From iteration 1 to 5, the global ear morphology became progressively smoother and more regularised, with better-defined helix and conchal folds and reduced residual asymmetries. Simultaneously, a gradual decrease in the global scale of the atlas was observed, consistent with the behaviour of the nearest-neighbour-based loss employed during registration. This shrinkage was interpreted as an inherent bias of the annotation-free correspondence formulation: template points are naturally pulled towards the interior of the population surface cloud rather than remaining on the peripheral manifold. We observed that this scale drift occurs consistently, indicating that it is primarily an effect of iterative mean-template updating under nearest-neighbour correspondence rather than a consequence of the scale-handling option alone. Importantly, after convergence, isotropic rescaling was applied to ensure that the final atlas height and width matched the training-set mean. This step ensured that subsequent statistical shape analysis and model-derived measurements remained physically interpretable and anatomically accurate. Overall, Figure 3 and Figure 4 jointly demonstrate that the iterative atlas construction reached convergence, and that superior alignment was consistently achieved by the non-rigid BiTPS pipeline compared to rigid ICP alone.

### 4.2. Agreement Between SSM-Derived and Manual Ear Dimensions

**Figure 5:**
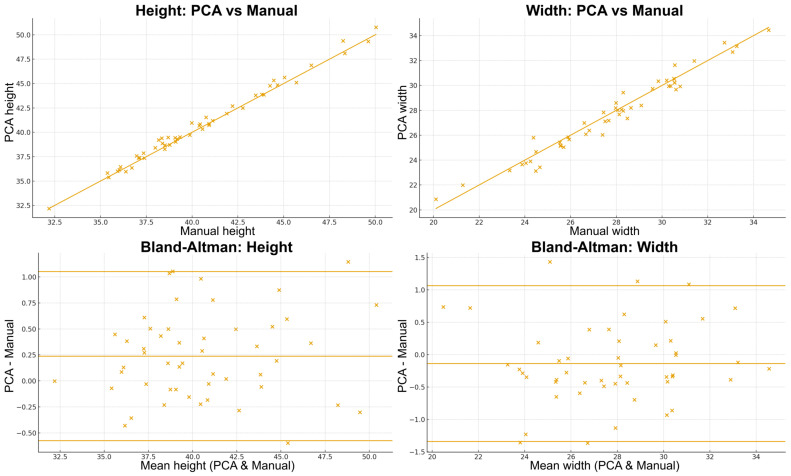
Agreement between SSM-derived and manual ear dimensions. (**Top row**): scatter plots of PCA-derived versus manual bowl height (**left**) and width (**right**), with identity lines indicating perfect agreement. (**Bottom row**): Bland–Altman plots for height (**left**) and width (**right**), showing the mean difference (solid horizontal line) and the LoA (mean ±1.96 SD).

SSM-derived bowl height and width were compared with the corresponding manual measurements obtained on the 3D meshes in *Geomagic Design X*. Across all 50 ears, an excellent correlation between PCA-derived height and manual height was observed (Pearson r=0.995, $p\approx 4.2\times 10^{-49}$; Spearman ρ=0.987, $p\approx 1.0\times 10^{-39}$), and a similarly strong association between PCA-derived width and manual width was observed (Pearson r=0.981, $p\approx 1.3\times 10^{-35}$; Spearman ρ=0.968, $p\approx 2.0\times 10^{-30}$), as illustrated by the scatter plots in the top row of Figure 5 and summarised in Table 2.

To evaluate potential systematic bias, the measurement difference for each ear was defined as the PCA-derived value minus the manual measurement, and paired *t*-tests were performed. For height, the mean difference was 0.24±0.41 (mean ± SD; same units as the original measurements), ranging from −0.60 to 1.14. A paired *t*-test indicated a statistically significant positive bias (t=4.06, p=0.00018; 95% confidence interval [0.12,0.35]), with PCA-derived heights being on average slightly larger than manual measurements. For width, the mean difference was −0.14±0.61 (range −1.36 to 1.43), and no significant systematic bias was observed (t=−1.59, p=0.12; 95% confidence interval [−0.31,0.03]).

Excellent agreement was further confirmed by Bland–Altman analysis (Figure 5, bottom row). For height, the mean difference was 0.24, with 95% LoA ranging from −0.57 to 1.05 (mean ±1.96 SD). For width, the mean difference was −0.14, with LoA ranging from −1.34 to 1.06. Most observations lay within these limits, and no obvious trend across the range of magnitudes was observed.

Overall agreement was further quantified using the ICC based on a two-way mixed-effects model for absolute agreement, single measurement (ICC(A,1)). The ICC was 0.993 for height and 0.980 for width. According to standard reliability guidelines, these values indicate excellent agreement, demonstrating that manual measurements can be reliably reproduced by the automated SSM-derived dimensions.

### 4.3. Additional Descriptive Morphometrics

In addition to linear dimensions that can be directly validated against manual measurements, several fully automatic 3D geometric descriptors are computed on the aligned atlas representation as descriptive statistics to facilitate interpretation of population-level variability. Table 3 summarises the distribution of the principal linear dimensions by PCA, and complementary descriptors including surface area and projection-based thickness proxies. For 3D descriptors, surface area is computed as total mesh area. Projection-based thickness proxies summarise the spread of vertex projections onto the third PCA axis of the mean shape. It is the distance from the deepest point of the concha to a plane defined by the auricular ridges (antihelix/tragus). This is a very practical way to measure “depth” for an engineer. These quantities provide additional context for understanding variation within the learned shape space.

### 4.4. Population Variability Along Principal Modes

The variance explained by the first ten principal components (PCs) of the auricular bowl SSM is presented in Table 4. The first principal component (PC1) accounted for 21.6% of the total shape variance, with the first five PCs explaining 60.8% of the variance. The cumulative variance increased rapidly by the first ten modes, which together were found to explain 73.9% of the total variability in auricular bowl shape. With additional modes, the explained variance increased further, with the first 20 modes capturing nearly 87% of the total shape variation.

Visual inspection of the shape deformations along the primary modes revealed clear anatomical interpretations. Mode 1 was primarily associated with the overall vertical extent of the auricular bowl: positive scores corresponded to an elevated superior conchal rim and an elongated antihelix, resulting in a taller bowl morphology; conversely, negative scores were associated with reduced bowl height and a more inferiorly positioned superior antihelix. Mode 2 primarily captured the horizontal aperture of the bowl: positive scores were characterised by a widening of the cavum and cymba conchae and a flattened antihelix, yielding broader, more open bowls; negative scores produced a narrower, more constricted conchal entrance. Mode 3 captured variations in conchal floor depth and antihelical prominence, ranging from a deep bowl with a pronounced antihelix to a shallower bowl with a smoother, less defined contour. Higher-order modes described localized morphological features, such as the orientation of the intertragic notch and subtle undulations along the conchal rim.

**Figure 6:**
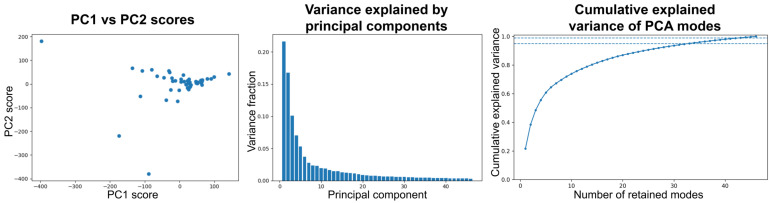
Population variability along principal components. (**Left**): scatter plot of subject scores on PC1 and PC2. (**Middle**): fraction of total variance explained by each principal component. (**Right**): cumulative explained variance as a function of the number of retained modes.

Taken together, these anatomically interpretable modes provide a compact basis for characterising individual differences in auricular bowl morphology that are directly relevant to contact area, acoustic sealing, and the mitigation of potential pressure hotspots in ear-related devices. This compactness indicates that population-wide variability in ear morphology can be effectively parametrised by a relatively small number of principal components, with higher-order modes capturing localized morphological variations. The distribution of subject scores (PC1 vs. PC2) and the variance explained by each mode are summarised in Figure 6. Figure 6 (right) further shows the cumulative explained variance as a function of the number of retained modes, providing an objective reference for selecting the number of modes in downstream reconstruction analyses.

### 4.5. Reconstruction Accuracy as a Function of SSM Dimensionality

To evaluate the number of principal components required to reconstruct individual ears with high geometric fidelity, we determined the lower bound of reconstruction error. Each aligned mesh was projected onto the SSM (built from all *N* = 50 ears) and reconstructed using the mean shape and the first *k* principal components. The vertex-wise root-mean-square error (RMSE) and the symmetric Chamfer-L_2_ distance between the original and reconstructed meshes were then computed to quantify the fidelity of the model.

**Figure 7:**
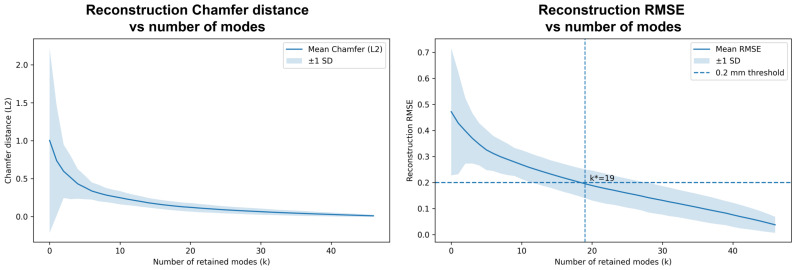
Reconstruction error as a function of the number of principal components (*k*) for 50 ears. (**Left**): mean symmetric Chamfer-L_2_ distance. (**Right**): mean vertex-wise RMSE, dashed horizontal line indicates the scanner resolution threshold (0.2 mm), yielding $k^{*}$ = 19 under the mean-RMSE criterion.

The mean reconstruction error across all 50 ears as a function of *k* is shown in Table 5 and Figure 7. Using the scanner resolution (∼0.2 mm) as an objective criterion, the minimum number of modes required to achieve mean RMSE ≤ 0.2 mm is $k^{*}=19$ (Figure 7, dashed lines). This provides a practical and application-relevant rule for selecting the model dimensionality, rather than arbitrarily retaining a large number of modes. When the mean shape alone was used (k=0), an average RMSE of 0.47 mm and a Chamfer-L_2_ distance of 1.00 mm^2^ were obtained. Accuracy was rapidly improved by the addition of a small number of modes: with k=5, the RMSE was reduced to 0.33 mm and the Chamfer-L_2_ distance to 0.38 mm^2^, corresponding to relative reductions of approximately 31% and 62%, respectively. With k=10, the mean RMSE was further reduced to 0.27 mm and the Chamfer-L_2_ distance to 0.25 mm^2^, and at k=20 the errors were decreased to 0.19 mm and 0.12 mm^2^. Beyond approximately 25–30 modes, a plateau was approached, with only modest gains despite increasing model dimensionality. When all 46 modes were used, a mean RMSE of 0.04 mm and a Chamfer-L_2_ distance of 0.01 mm^2^ were obtained.

These results indicated that a favourable trade-off between compactness and geometric fidelity was offered by the proposed SSM. In practice, sub-millimetre vertex accuracy and substantial reduction in global surface error are already provided by retaining 10–20 modes, while a low-dimensional parameter space is maintained and suitability for downstream analysis and device optimization is retained.

## 5. Discussion

An annotation-free pipeline for constructing a 3D ear atlas and statistical shape model of the auricular bowl was presented, based on iterative rigid and non-rigid registration. While rigid ICP, TPS/BiTPS, and PCA are established components, our contribution lies in integrating them into a fully automatic, annotation-free pipeline tailored to auricular bowl morphology, together with a transparent evaluation suite. In this context, the correlation between PCA-derived reconstructions and simple linear dimensions is not presented as a mathematical novelty of PCA, but as evidence that the learned shape space supports reproducible and interpretable measurements under a consistent correspondence framework. Using 50 auricular meshes, registration accuracy systematically improved over successive atlas updates. The proposed BiTPS refinement scheme consistently achieved lower registration errors and higher surface coverage than rigid ICP alignment alone. Across five outer iterations, all metrics approached a plateau, indicating that atlas construction was numerically stable and that further iterative re-averaging was not required for convergence.

The present framework significantly reduces the dependence on manually placed landmarks compared with previous ear and concha modelling approaches. Earlier studies on ear canals and auricular conchae typically relied on sparse anatomical landmarks or dense sets of manually defined control points to wrap a template mesh to each subject prior to statistical modelling [10,19]. While these methods demonstrated the feasibility of ear-related SSMs and provided valuable population data, the manual landmarking process is labour-intensive and susceptible to both inter- and intra-observer variability, particularly in regions with complex folds and ill-defined boundaries, such as the auricular bowl. In contrast, our pipeline establishes dense surface correspondence via a nearest-neighbour search on iteratively refined atlases followed by BiTPS refinement, requiring no manual point annotations. The curvature maps across iterations suggest that local geometric features such as the helical rim, antihelix, and conchal floor are preserved while global alignment is improved, further supporting the anatomical plausibility of the resulting mean ear.

The SSM derived from this atlas utilized a compact set of principal modes to capture auricular bowl variability, yielding geometric measurements that closely reproduced manual 3D mesh dimensions. Near-perfect correlations with reference values and excellent absolute agreement (ICC ≥0.98) were observed for PCA-derived bowl height and width, with Bland–Altman limits within approximately ±1 mm. The high ICC values and narrow LoA in the Bland–Altman analysis confirm the metrological interchangeability of the automated model with manual measurements, validating the ability of the registration pipeline to capture individual morphological variations.

While the agreement analysis validates measurement interpretability under a shared geometric definition, geometric fidelity and compactness are separately assessed by reconstruction errors and registration metrics. A small but statistically significant positive bias was observed for bowl height, whereas no detectable systematic offset was observed for width. These findings indicate that manual measurements in studies of ear morphology can be replaced by the SSM as a reliable surrogate, while the SSM also provides richer information on shape variation than that provided by a small set of linear distances. In this way, the present framework complements previous 3D ear anthropometry work, in which emphasis has mainly been placed on summarising linear dimensions and on classifying external ear shapes for product sizing and clustering [20,21].

Several limitations should be acknowledged. First, the sample size was relatively small (*N* = 50), and the study was restricted to young East Asian adults, which may limit the generalisability of the atlas to other age groups, ethnicities, and extreme morphologies. Larger, more diverse datasets will be required to quantify population variability more comprehensively and to derive population-specific atlases where necessary. Second, all data were acquired using a single 3D scanner, and the analysis was restricted to the auricular bowl region. Consequently, the ear canal and other peri-auricular structures—which are also critical for acoustic performance and device retention—were not modelled. Third, the correspondence model was purely geometric; it did not incorporate the mechanical properties of auricular cartilage or soft-tissue compliance under load, both of which significantly influence perceived comfort and pressure-discomfort thresholds [9]. Finally, while the SSM was validated against manual morphological measurements, a direct linkage between SSM-derived descriptors and functional outcomes—such as acoustic response, stimulation thresholds, or subjective comfort ratings—remains to be established.

We further evaluated sensitivity to the number of FPS/TPS control points (m=1000/2500/5000) and the BiTPS regularisation strength ($\lambda =10^{-3}$–$10^{-1}$). Registration metrics and reconstruction curves showed only marginal and modest changes, and the scanner-resolution-based choice of model dimensionality remained stable ($k^{*}=18$–19). The number of control points (m=1000) was selected based on a sensitivity analysis confirming that higher sampling densities did not significantly improve registration fidelity or model compactness (see Supplementary Material S1). The regularisation parameter λ was optimized to ensure that the maximum surface deviation between the original scan and the registered mesh did not exceed the hardware resolution of 0.2 mm, thereby preserving fine-scale anatomical features. More specifically, see Supplementary S1 (Table S1.1 and Figures S1.1–S1.3) and Supplementary S2 (Table S2.1 and Figures S2.1 and S2.2). Finally, we assessed the out-of-sample generalization of the model using five-fold cross-validation. As shown in Supplementary S3, the test-set reconstruction curves show a consistent error reduction with diminishing returns beyond k≈24. The mean test reconstruction RMSE for held-out samples decreased to 0.386 mm at k=37, confirming the model’s high fidelity in capturing previously unseen auricular surface undulations.

## 6. Conclusions

A fully annotation-free framework was developed and validated for constructing a 3D ear atlas and statistical shape model (SSM) of the auricular bowl from surface scans. By combining iterative atlas construction with rigid ICP and non-rigid BiTPS registration, the proposed pipeline established dense surface correspondence without the need for manual landmarks, yielding a numerically stable population-mean ear. Across five atlas iterations, registration errors were substantially reduced, and surface coverage approached completeness for both rigid and non-rigid stages. The BiTPS refinement consistently outperformed rigid-only registration, ensuring high geometric fidelity and a robust basis for modelling auricular morphology.

The resulting SSM captures the inter-individual variability of the auricular bowl within a compact set of principal modes, reproducing manual 3D mesh measurements of height and width with excellent agreement. These results demonstrate that an annotation-free, surface-based modelling approach provides geometrically interpretable descriptors suitable for replacing or complementing traditional linear anthropometry. Consequently, this framework offers a robust, automated alternative for high-fidelity ear morphology analysis in both ergonomic design and clinical applications.

Methodologically, this framework offers a generic, annotation-free strategy for atlas and SSM construction that can be extended to other anatomical structures represented as 3D surfaces. From an application perspective, the auricular atlas and SSM provide a robust geometric foundation for the design and optimization of medical and wearable devices. Furthermore, this model facilitates the refinement of auricular mapping schemes used in neuromodulation and auricular therapy. Future work will focus on larger, more diverse populations and the inclusion of full-pinna and ear-canal geometries. By integrating these morphological models with biomechanical and functional evaluations, 3D shape characteristics can be more directly linked to device performance, stimulation efficacy, and subjective user comfort.

## Figures and Tables

**Table 1:** Mean registration metrics at the first and fifth atlas iterations for rigid ICP and non-rigid BiTPS (50 ears).

Metric	ICP (Rigid)		BiTPS (Elastic)	
	Iteration 1	Iteration 5	Iteration 1	Iteration 5
mean_nn	2.10	0.93	0.71	0.30
max_nn	8.92	2.97	3.73	1.32
Chamfer-L_2_	13.46	5.47	1.47	0.71
${\mathrm{Coverage}}_{\tau }$	0.61	0.89	0.92	0.98

**Table 2:** Agreement statistics between SSM-derived and manual ear dimensions (50 ears). The difference is defined as PCA minus manual. LoA: Bland–Altman limits of agreement (mean ±1.96 SD).

Dimension	Pearson *r*	Spearman ρ	Mean Diff	SD Diff	LoA Lower	LoA Upper	ICC(A,1)
Height	0.995	0.987	0.24	0.41	−0.57	1.05	0.993
Width	0.981	0.968	−0.14	0.61	−1.34	1.06	0.980

**Table 3:** Descriptive morphometrics computed on the aligned atlas representation (50 ears). The upper block reports principal linear dimensions; lower block reports additional 3D geometric descriptors for interpretability. Values are summary statistics across subjects.

Metric	Mean	SD	Median	Min	Max	IQR
*Linear dimensions*						
height_e1	40.72	4.03	39.62	32.19	50.76	5.57
width_e2	27.70	3.12	27.90	20.85	34.45	4.46
thickness_e3	17.78	1.57	17.99	14.51	21.62	1.95
ratio_e1_e2	1.48	0.14	1.49	1.24	1.78	0.17
*Additional 3D descriptors*						
Surface area	1551.73	324.40	–	1419.53	1733.10	–
Projection range	10.67	1.96	–	10.04	11.61	–

**Table 4:** Variance explained by the leading principal components of the auricular bowl SSM.

PC Index	Variance Explained (%)	Cumulative Variance (%)
1	21.6	21.6
2	16.8	38.4
3	10.1	48.5
4	7.0	55.6
5	5.3	60.8
6	3.7	64.6
7	2.8	67.3
8	2.3	69.7
9	2.3	72.0
10	1.9	73.9
20	0.9	87.0

**Table 5:** Mean reconstruction error as a function of the number of retained principal components (*k*) for 50 ears.

Number of Modes *k*	Mean RMSE (mm)	Mean Chamfer-L_2_ (mm^2^)
0	0.47	1.00
5	0.33	0.38
10	0.27	0.25
20	0.19	0.12
30	0.13	0.06
46	0.04	0.01

## Data Availability

Due to privacy and ethical considerations the data presented in this study may only be made available from the corresponding author upon reasonable request and subject to approval by the institutional ethics committee.

## Supplementary Materials

The following supporting information can be downloaded at: https://www.mdpi.com/article/10.3390/s26113493/s1, Supplementary S1: Sensitivity to the number of FPS/TPS control points *m*; Table S1.1: Sensitivity to the number of FPS in BiTPS control points (m): mean registration metrics after the final atlas iteration (50 ears). Values are mean across subjects; Figure S1.1: Sensitivity to the number of control points *m*: mean reconstruction RMSE versus the number of retained modes *K*. The dashed line indicates the 0.2 mm scanner-resolution threshold; $k^{*}$ denotes the smallest *k* achieving mean RMSE less than or equal to 0.2 mm for each setting; Figure S1.2: Sensitivity to the number of control points *m*: mean Chamfer-L_2_ distance versus the number of retained modes *K*; Figure S1.3: Distribution of automatically selected FPS pseudo-landmarks on the atlas template for different numbers of control points (m=1000/2500/5000). Increasing m densifies surface coverage, while the final atlas geometry remains visually consistent; Supplementary S2: Sensitivity to the BiTPS regularization strength (*λ*); Table S2.1: Sensitivity to the BiTPS regularization strength (λ): mean registration metrics after the final atlas iteration (50 ears). Values are mean across subjects; Figure S2.1: Sensitivity to BiTPS regularization (λ): mean reconstruction RMSE versus the number of retained modes *K*. The dashed line indicates the 0.2 mm scanner-resolution threshold; $k^{*}$ denotes the smallest *k* achieving mean RMSE less than or equal to 0.2 mm for each setting; Figure S2.2: Sensitivity to BiTPS regularization (λ): mean Chamfer-L_2_ distance versus the number of retained modes *K*; Supplementary S3: Five-fold cross-validation evaluation; Figure S3.1: Five-fold cross-validation: out-of-sample reconstruction RMSE versus the number of retained modes *k*. Shaded region indicates ±1 SD across folds; Table S3.1: Five-fold cross-validation (test set) reconstruction RMSE as a function of the number of retained modes *k*. Values are mean ± SD across folds; Table S3.2: Five-fold cross-validation: mean registration metrics on the test sets after mapping to the training-fold atlas (10 ears per fold). Values are mean ± SD across folds.

## Abbreviations

The following abbreviations are used in this manuscript:

3D	Three-Dimensional
AEP	Anterior Extremal Point
AP	Anterior–Posterior
BiTPS	Bidirectional Thin-Plate Spline
CI	Confidence Interval
FPS	Farthest Point Sampling
ICC	Intraclass Correlation Coefficient
ICP	Iterative Closest Point
IEP	Inferior Extremal Point
KD-tree	K-dimensional Tree
LoA	Limits of Agreement
NN	Nearest-neighbour
PC	Principal Component
PCA	Principal Component Analysis
PEP	Posterior Extremal Point
RMSE	Root-Mean-Square Error
ROI	Region of Interest
SD	Standard Deviation
SEP	Superior Extremal Point
SI	Superior–Inferior
SSM	Statistical Shape Model
STL	Stereolithography (file format)
SVD	Singular Value Decomposition
taVNS	Transcutaneous Auricular Vagus Nerve Stimulation
TCM	Traditional Chinese Medicine
TPS	Thin-Plate Spline
