# Supplementary material for: An Annotation-Free Pipeline for 3D Auricular Bowl Atlas Construction and Statistical Shape Modelling from Surface Scans
Source: Sensors (Basel). 2026 Jun 1;26(11):3493. doi: 10.3390/s26113493 (PMC13259287; doi:10.3390/s26113493)
Supplement: Supplementary file 1 [file sensors-26-03493-s001.zip › sensors-4174073-supplementary.pdf]

Supplementary S1: Sensitivity to the number of FPS/TPS control points *m*

To assess robustness to the number of automatically selected pseudo-landmarks used to parameterize the BiTPS deformation field, the atlas/SSM pipeline with *m* in 1000,2500,5000 control points was repeated, and all other settings fixed.

Table S1.1 summarizes registration quality after the final atlas iteration. Across this range, alignment metrics changed only marginally: mean_nn decreased from 0.340 (m = 1000) to 0.313 (m = 5000), while Coverage_ τ remained essentially unchanged, indicating near-complete atlas coverage.

Figure S1.1 shows reconstruction RMSE as a function of the number of retained PCA modes K for each m. Using the scanner-resolution criterion (0.2 mm) to select the smallest practical dimensionality, the resulting k^* values were stable (k^* = 18 for m = 1000 and k^* = 19 for m = 2500/5000).

Figure S1.2 reports the corresponding Chamfer_L2 curves, which exhibit the same trend. Together, these results indicate that our reconstruction conclusions are not sensitive to the choice of *m* within a reasonable range.

We additionally visualized the spatial distribution of FPS pseudo-landmarks for different control-point counts (m = 1000/2500/5000).

As shown in Figure S1.3, increasing *m* densifies surface coverage, while atlas geometry and reconstruction conclusions remain stable.

Table S1.1 Sensitivity to the number of FPS in BiTPS control points (m): mean registration metrics after the final atlas iteration (50 ears). Values are mean across subjects.

m	mean_nn	max_nn	Chamfer_L2	Coverage_ τ	n
1000	0.34	1.31	1.21	1.00	50
2500	0.32	1.29	1.01	1.00	50
5000	0.31	1.30	0.82	1.00	50

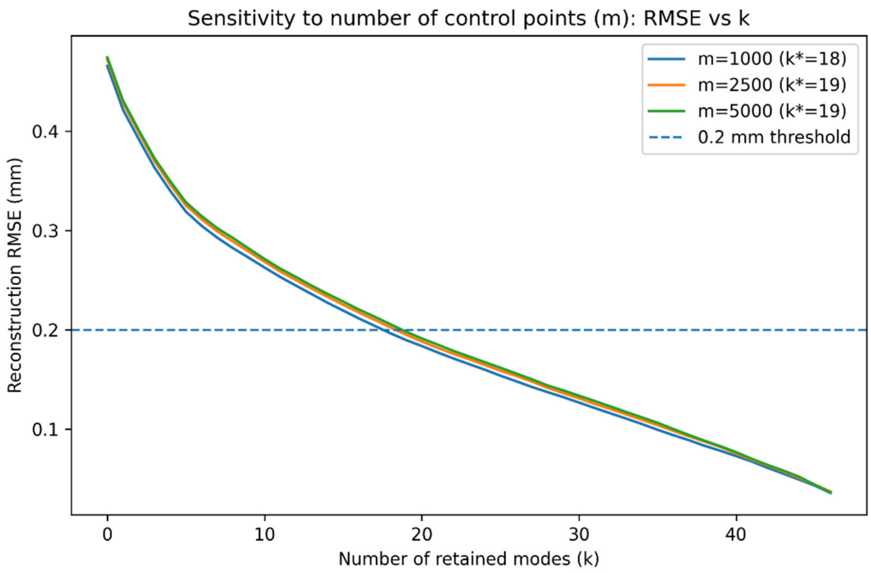

Figure S1.1 Sensitivity to the number of control points *m*: mean reconstruction RMSE versus the number of retained modes *K*. The dashed line indicates the 0.2 mm scanner-resolution threshold, k^*denotes the smallest *k* achieving mean RMSE $\leqslant$ 0.2 mm for each setting.

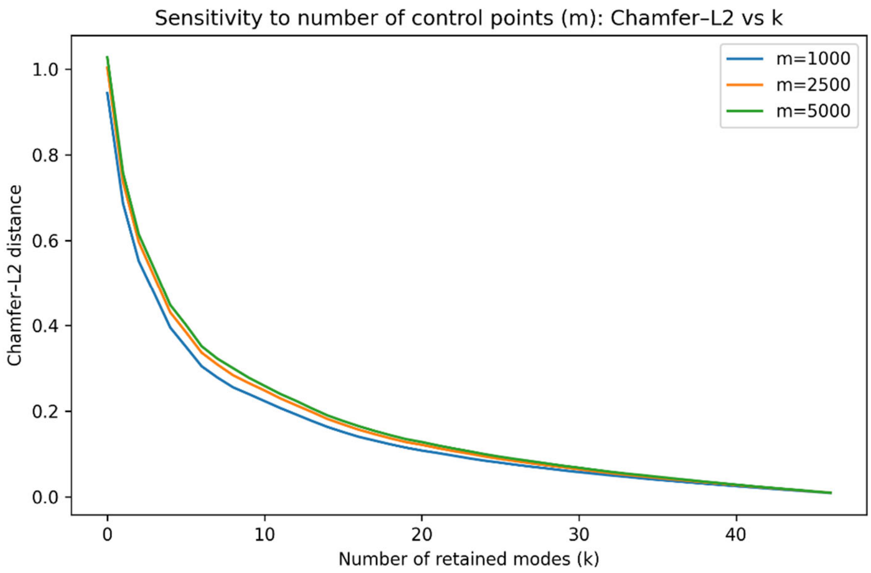

Figure S1.2 Sensitivity to the number of control points *m*: mean Chamfer_L2 distance versus the number of retained modes *K*.

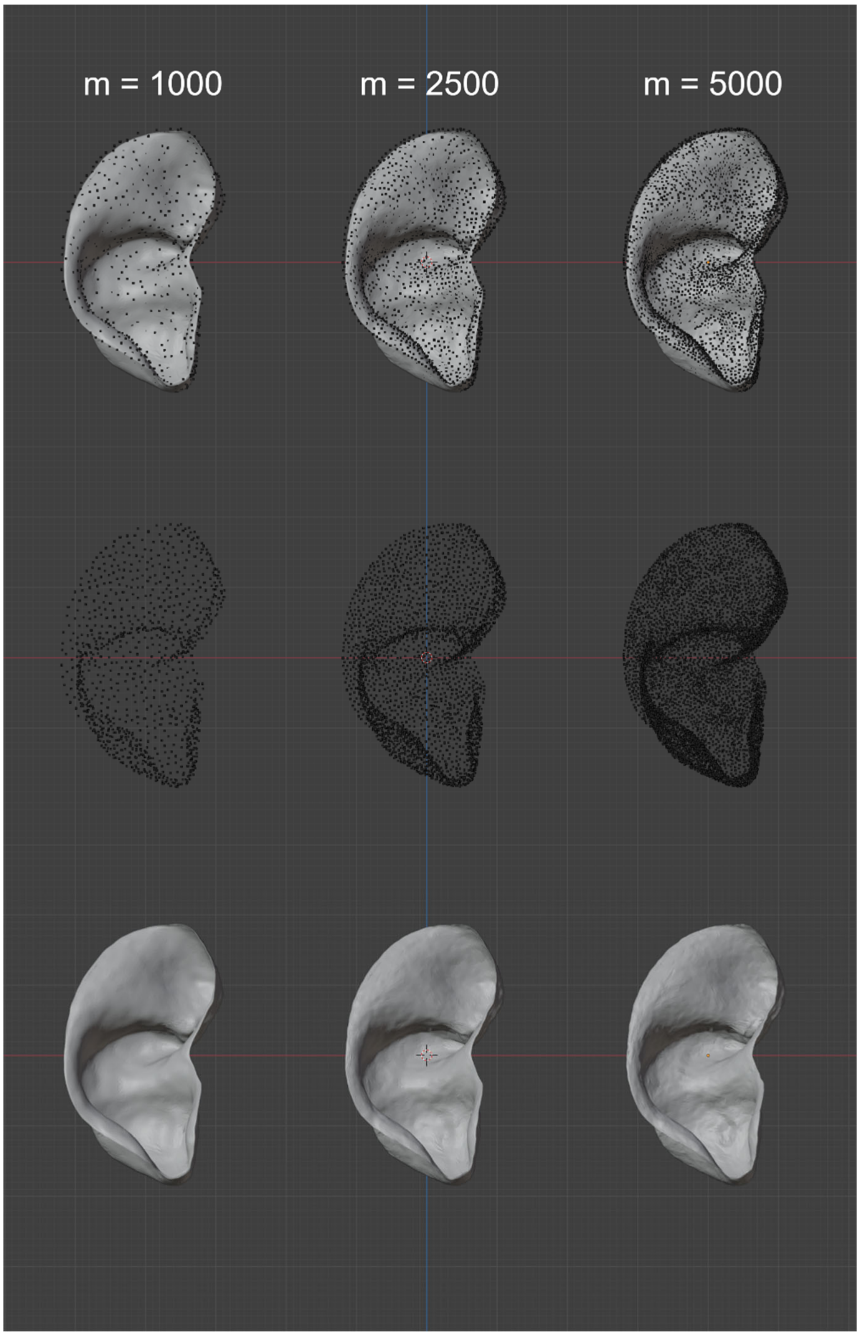

Figure S1.3 Distribution of automatically selected FPS pseudo-landmarks on the atlas template for different numbers of control points (*m* = 1000/2500/5000). Increasing *m* densifies surface coverage, while the final atlas geometry remains visually consistent

Supplementary S2: Sensitivity to the BiTPS regularization strength (λ)

To assess robustness to the BiTPS smoothness regularization, we repeated the atlas/SSM pipeline with three values of the regularization strength, $\lambda \in \{10^{-3}, 10^{-2}, 10^{-1}\}$, while keeping all other settings fixed (including $m = 2500$ control points).

Table S2.1 summarizes registration quality after the final atlas iteration. Across this range, the metrics remain highly stable: mean_nn stays near 0.32 and Coverage_τ remains ≈0.999, indicating near-complete atlas coverage. Chamfer_L2 also changes only modestly across λ.

Figure S2.1 shows reconstruction RMSE versus the number of retained PCA modes k for each λ. Using the scanner-resolution criterion (0.2 mm) to select the smallest practical dimensionality, the resulting k* values were stable ($k^* = 19$ for $\lambda = 10^{-3}$ and 10^{-2}, and $k^* = 18$ for $\lambda = 10^{-1}$).

Figure S2.2 reports the corresponding Chamfer_L2 curves. Overall, the learned shape space and the reconstruction conclusions are robust to the BiTPS regularization strength within a reasonable range.

Table S2.1 Sensitivity to the BiTPS regularization strength (λ): mean registration metrics after the final atlas iteration (50 ears). Values are mean across subjects.

λ	mean_nn	max_nn	Chamfer_L2	Coverage_ τ	n
1e-3	0.32	1.32	1.03	1.00	50
1e-2	0.32	1.29	1.01	1.00	50
1e-1	0.32	1.31	0.97	1.00	50

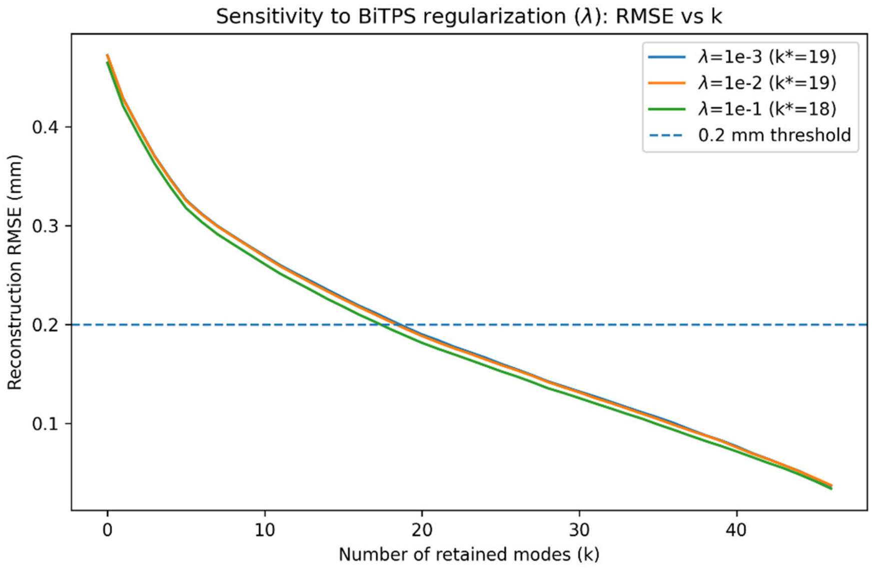

Figure S2.1. Sensitivity to BiTPS regularization (λ): mean reconstruction RMSE versus the number of retained modes *K*. The dashed line indicates the 0.2 mm scanner-resolution threshold; k^* denotes the smallest k achieving mean RMSE ⩽ 0.2 mm for each setting.

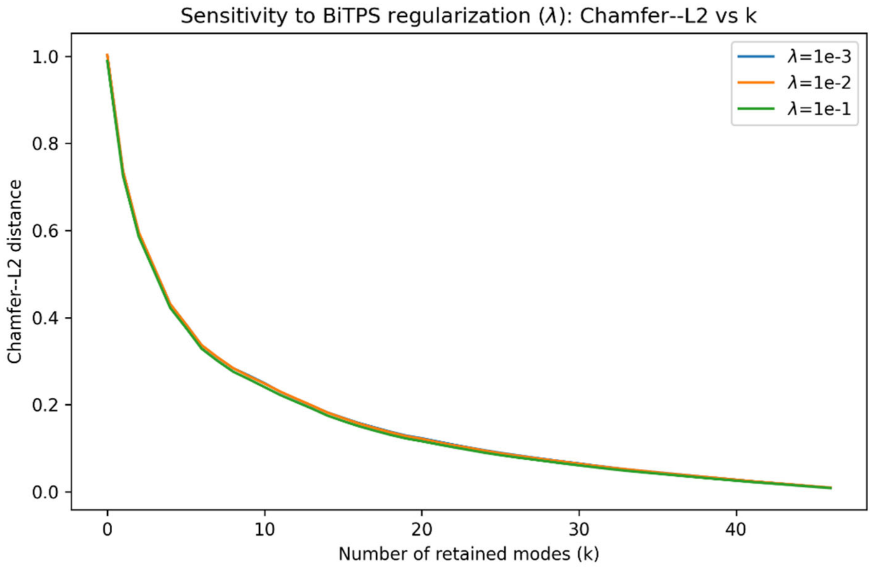

Figure S2.2. Sensitivity to BiTPS regularization (λ): mean Chamfer_L2 distance versus the number of retained modes *K*.

Supplementary S3: Five-fold cross-validation evaluation

To assess out-of-sample generalization and address concerns about in-sample reconstruction, we performed five-fold cross-validation. In each fold, the atlas and PCA modes were learned from the training subset (40 ears), and reconstruction RMSE was evaluated on the held-out test subset (10 ears) using the training-fold mean shape and modes.

Figure S3.1 reports the aggregated test reconstruction curve (mean ± SD across folds). Test RMSE decreases monotonically from 0.518 at k=0 (mean shape only) to 0.386 at k=37. A clear diminishing-return regime is observed beyond approximately $k \approx 24$, consistent with the elbow of the cross-validated curve.

Key values are listed in Table S3.1. Table S3.2 further summarizes registration quality on the held-out test sets after mapping to the training-fold atlas.

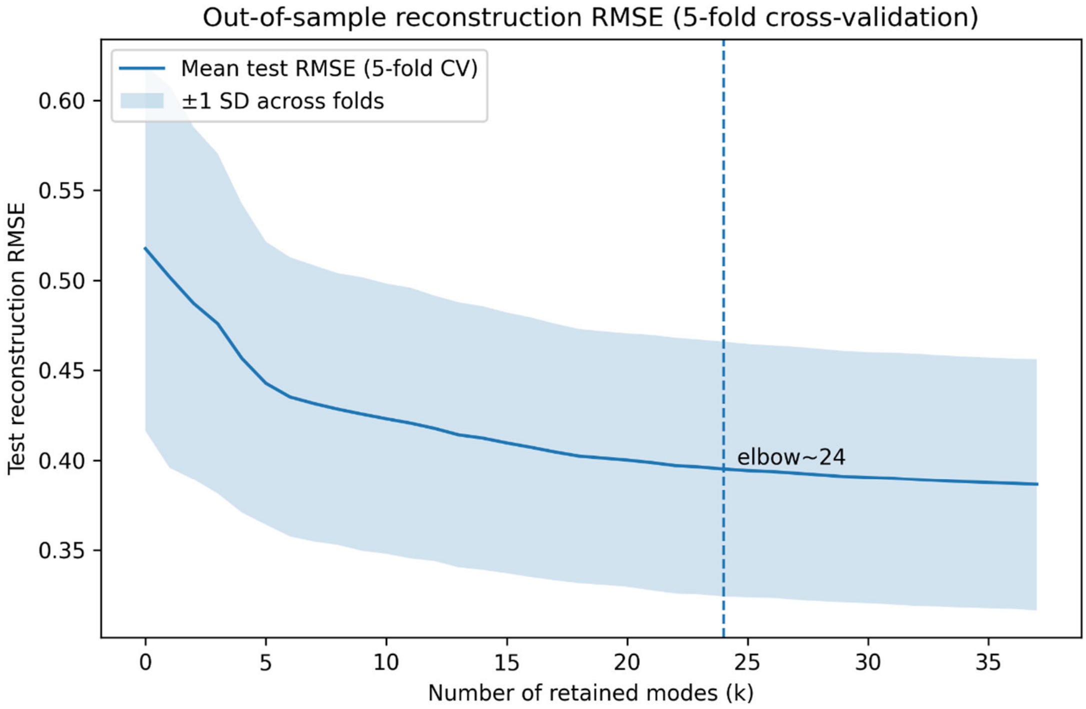

Figure S3.1. Five-fold cross-validation: out-of-sample reconstruction RMSE versus the number of retained modes k. Shaded region indicates ±1 SD across folds.

Table S3.1 Five-fold cross-validation (test set) reconstruction RMSE as a function of the number of retained modes k. Values are mean ± SD across folds.

k	Mean test RMSE	SD (across folds)	n_folds
0	0.518	0.101	5
24	0.395	0.071	5
37	0.386	0.070	5

Table S3.2 Five-fold cross-validation: mean registration metrics on the test sets after mapping to the training-fold atlas (10 ears per fold). Values are mean ± SD across folds.

Metric	mean_nn	max_nn	Chamfer_L2	Coverage_τ
Mean ± SD	0.314 ± 0.016	1.296 ± 0.111	1.129 ± 0.505	0.9987 ± 0.0018
